# Micro-CT Evaluation of Four Root Canal Obturation Techniques

**DOI:** 10.1155/2021/6632822

**Published:** 2021-02-25

**Authors:** Mahmood Reza Kalantar Motamedi, Amin Mortaheb, Maryam Zare Jahromi, Brett E. Gilbert

**Affiliations:** ^1^Department of Endodontics, School of Dentistry, Isfahan (Khorasgan) Branch, Islamic Azad University, Isfahan, Iran; ^2^Department of Endodontics, College of Dentistry, University of Illinois at Chicago, Chicago, IL, USA

## Abstract

**Purpose:**

The aim of this *in vitro* study was to evaluate the quality of four root canal obturation techniques using microcomputed tomography (micro-CT).

**Materials and Methods:**

A total of 36 mandibular first premolars with mostly round canals were decoronated, then instrumented up to a size F3 rotary file, and dressed with an epoxy resin-based sealer. Subsequently, they were divided into 4 different groups (*n* = 9) based on the method of obturation: lateral condensation using 0.02 tapered master cone (LC2), lateral condensation using 0.04 tapered master cone (LC4), matched single-cone technique (MS), and matched single cone-mediated ultrasonic activation (MSUA). All the teeth were scanned using micro-CT (resolution of 19 *μ*m), and the percentage volume of voids was calculated. One-way analysis of variance and Tukey test were used to analyze the data (*α* = 0.05).

**Results:**

The total percentage volume of voids was significantly lower in the MSUA group compared to all other groups (*P* < 0.05). The total percentage volume of voids was significantly lower in the MS group compared to the LC4 (*P* < 0.001) and LC2 (*P* < 0.001) groups. However, there was no significant difference between the LC2 and LC4 groups (*P* < 0.65).

**Conclusions:**

MSUA, significantly, showed the least root canal filling voids amongst all the obturation techniques studied. MSUA can be considered an effective method for the filling of the round root canals. In general, lateral condensation using either 0.02 or 0.04 tapered master cones had significantly the highest volume percentage of voids amongst the experimental groups.

## 1. Introduction

An ideal root canal filling should three dimensionally and homogenously obliterate the root canal system [1]; however, most completed root canal fillings are not fully able to do so [2]. The unfilled spaces within the root canal system may lead to some complications, because they might be comprised of remaining microorganisms that can be nourished and multiply when they come in contact with lateral canals and the periapical region [3].

There are several root canal obturation methods; cold lateral condensation is the most widely used technique [4]. This technique is considered a reference for evaluating other obturation techniques. It is a low-cost and simple technique. The conventional cold lateral technique involves fitting of an ISO-standardized master gutta-percha (GP) cone with an apical to coronal taper of 0.02, followed by lateral condensation using spreaders and fitting of additional accessory cones with sealer interspersed throughout [5] However, accessory GP cones used in this technique cannot fully be adapted to the root canal walls and irregularities. Therefore, void occurrence between the individual cones and the root canal walls is probable [6]. However, if a dentist uses 0.06 or even more tapered rotary or reciprocal instrument, carrying out the lateral condensation technique using an ISO-standardized master cone might require numerous accessory cones to closely match the prepared space. This procedure is time-consuming and may also lead to further voids. In order to overcome these drawbacks, the operator may choose a more tapered (e.g., 0.04 or 0.06) master cone more closely to the size and taper of the final shaping instrument [7].

In a matched single-cone technique, a single master GP cone corresponding to the size and taper of the final shaping instrument is introduced to the root canal in conjunction with a sealer. It is claimed that the precise fit of GP creates a hydraulic pressure that pushes the sealer laterally into the irregularities and dentinal tubules [8]. Although it is a simple technique and decreases the working time, it has its potential limitations, especially the increase of sealer thickness in oval canals which can result in dimensional shrinkage during the setting of the sealer [4]. It is suggested to take advantage of sealers with good physical and chemical characteristics, e.g., high flowability, adaptation to the irregularities of the root canal system, and a low volume of shrinkage [9]. Moreover, based on two microcomputed tomography (micro-CT) studies, it is suggested to apply ultrasonic energy to better facilitate a thorough distribution of EndoSeal MTA, a premixed calcium silicate sealer [10, 11]. However, this method has not been evaluated by the aid of micro-CT when using resin-based sealers for a matched single-cone technique. The authors of this study could not find any micro-CT studies to compare the quality of lateral condensation root canal fillings when using 0.02 and 0.04 tapered master cones. Therefore, the aim of the present study is to evaluate the quality of four root canal obturation techniques with an epoxy resin-based sealer: (1) lateral condensation using 0.02 tapered master cone, (2) lateral condensation using 0.04 tapered master cone, (3) matched single-cone technique, and (4) matched single cone-mediated ultrasonic activation.

## 2. Materials and Methods

### 2.1. Sample Selection and Preparation

The present study was approved by the Ethics Committee and Institutional Review Board of the University in Jun 15, 2020 (Ethical code: IR.IAU.KHUISF.REC.1399.062). A total of 36 human mandibular first premolar, which were extracted due to orthodontic or periodontal reasons, were selected. The teeth were stored in sterile distilled water until commencement of the study. On the day of the study, the teeth were immersed in a 5.25% sodium hypochlorite (NaOCl) for 30 minutes.

Teeth with immature apices, previous root canal treatment, root caries or restorations, and root resorptions were excluded from the study. Moreover, the teeth with initial apical foramen size of #20 or greater, as verified by a K-file (Mani Inc., Tochigi-Ken, Japan), were excluded after access cavity preparation. Only teeth with straight root canals were included based on Schneider's method [12].

Prior to the start of the experiment, preoperative radiographs were taken to evaluate the anatomy of the teeth. The round canal criteria were dependent on the anatomy fitting the following formula. If in the middle to apical third of the root canal system the internal long : short diameter of the root canal was <2, it was considered a nonoval root canal system [13]. Only specimens with mostly round canals and Vertucci's type I were selected [14, 15].

### 2.2. Root Canal Preparation

All the teeth were decoronated using a high-speed handpiece and cylindrical diamond bur to obtain a specimen size of 14 mm. Access into the canals was carried out, and working length was determined by introducing a #10 K-file (Mani Inc.) into the canal until it was just visible at the apical foramen. The working length was set at 0.5 mm short of that length. Glide path was established using a #15 K-file (Mani Inc.). The roots were instrumented to size the F3 file (PathMax Pro, Nikinc Dental, Eindhoven, The Netherlands). This file has similar geometric characteristics to ProTaper® and ProTaper® Gold with a tip size of 0.30 mm with varying degrees of taper: 9% from D0 to D3, 6% from D3 to D5, and a progressively reducing taper (5-3%) from D5 to D16. During preparation, a copious amount of 5.25% sodium hypochlorite and a side-venting needle (30-Gauge, Cerkamed Medical Company, Stalowa, Poland) were used. Patency was also maintained using a #10 K-file 0.5 mm beyond the apex between each rotary file. The final irrigation of the samples was completed using 2 mL 17% EDTA (MD Cleanser, Meta Biomed, Chungju, Korea) for 1 min, followed by 2 mL 5.25% NaOCl and rinsing with 2 mL saline solution.

### 2.3. Root Canal Obturation

After completion of instrumentation, the canals were dried with sterile paper points (DiaDent, Chongju, Korea). The samples were randomly assigned to four groups (*n* = 9) according to the filling technique used.

#### 2.3.1. Group 1: Lateral Condensation using 0.02 Tapered Master Cone (LC2)

Standard lateral condensation technique was carried out using standard 0.02 taper GP [16]. Each master cone demonstrated tug-back. Prior to the insertion of the master cone, AdSeal sealer (Meta BioMed) was placed inside the canal space using Lentulo-spiral (#30, Mani Inc.). Then, an ISO-standard master cone (#30, Meta BioMed) was coated with the sealer and inserted into the canal to the working length. Finger spreaders (#25 or #30, Mani Inc.) were introduced between the master cone and dentinal walls to make room for accessory cones. This procedure was repeated until the spreader could not penetrate more than 2-3 mm into the canal orifice; the obturation was completed. The excess GP was then removed with an electrical heat carrier (E&Q Plus, Meta BioMed) 1 mm below the orifice and vertically compacted with a cold plugger.

#### 2.3.2. Group 2: Lateral Condensation using 0.04 Tapered Master Cone (LC4)

The procedure was the same as the previous group (LC2), except for the use of 0.04 taper GP as the master cone (0.04 taper, #30, Meta BioMed).

#### 2.3.3. Group 3: Matched Single-Cone Technique (MS)

AdSeal was placed into the root canals using a Lentulo-spiral. An F3 master GP cone (Dentsply Maillefer, Ballaigues, Switzerland) coated with the sealer was slowly inserted into the canal to its working length. Then, the excess GP was trimmed off with an electrical heat carrier 1 mm below the orifice and vertically compacted with a cold plugger.

#### 2.3.4. Group 4: Matched Single Cone-Mediated Ultrasonic Activation (MSUA)

**Figure 1:**
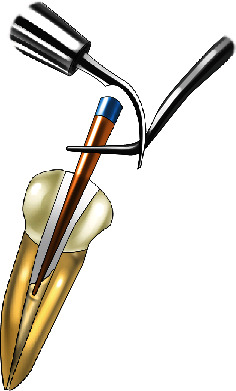
Matched single-cone by ultrasonic activation with AdSeal (previously inserted into the root canal with the aid of a Lentulo-spiral).

An ultrasonic tip was connected to an ultrasonic device (Woodpecker/DTE D5, Woodpecker Medical Instrument, Guilin, Guangxi, China), which was set on “G” mode (i.e., the moderate power of the device). After placing the AdSeal sealer into the canal with a Lentulo-spiral, ultrasonic vibration was applied to a locking cotton plier that held the F3 GP cone 18 mm from the tip. The cone coated with the sealer was slowly inserted into the working length during continuous ultrasonic activation (Figure 1). The ultrasonic application time during GP cone placement was 3 s. This method was replicated from the study of Kim et al. [11]. Then, the excess GP was cut off with an electrical heat carrier 1 mm below the orifice and vertically compacted with a cold plugger.

In all the groups, the access openings were sealed using a bonding agent (G-Premio bond, GC Corp., Tokyo, Japan) and flowable composite resin (Beautifil Flow, Shofu Inc., Kyoto, Japan). The teeth were then stored at a relative humidity of 100% and 37°C for 7 days.

### 2.4. Micro-CT and Image Analysis

In this study, a micro-CT scanner (LOTUS-inVivo, Behin Negareh Co., Tehran, Iran) was used [17]. LOTUS-inVivo has a cone-beam micro-focus X-ray source and a flat panel detector. In order to obtain the best possible image quality, the X-ray tube voltage and current were set at 80 kV and 100 *μ*A, respectively. The resolution was 19 *μ*m. Total scan duration was 28 minutes.

All the protocol settings process was controlled using LOTUS-inVivo-ACQ software. The acquired 3D data was reconstructed using LOTUS inVivo-REC by a standard Feldkamp, Davis, Kress (FDK) algorithm [18], by which quantification of the root canal volume, filling material volume (GP/sealer), and void volume was carried out.

The percentage volume of voids was calculated by using the following formula:

(1) $$\begin{array}{c} \text{Voids volume}\left(\%\right)=\frac{\text{Void volume}}{\text{Canal volume}}*100. \end{array}$$

All the analyses were conducted separately for the coronal, middle, apical thirds (3 mm each) and the whole of the root canal obturation.

### 2.5. Statistical Analysis

The data were entered into the Statistical Package for the Social Sciences (SPSS) (SPSS Inc., Version 21, Chicago, USA) and statistically analyzed using the Kolmogorov-Smirnov test for determination of normal distribution and then followed by one-way analysis of variance and Tukey tests to detect any significance (*α* = 0.05).

## 3. Results

The mean volume percentage of voids and significant differences are shown in Table 1.

Overall, the canals obturated using MSUA showed the lowest percentage volume of voids compared to the other groups (*P* < 0.05). The mean volume percentage of voids in the MS group was statistically lower than that in the LC4 group (*P* < 0.001) and LC2 group (*P* < 0.001), whereas there was no significant difference between the LC4 and LC2 groups (*P* < 0.65).

In the coronal third, the canals obturated using MSUA showed the lowest percentage volume of voids compared to the other groups (*P* < 0.05). The mean volume percentage of voids in the MS group was statistically lower than that in the LC4 group (*P* < 0.001) and LC2 group (*P* < 0.001), whereas there was no significant difference between the LC4 and LC2 groups (*P* < 0.97).

In the medial third, the canals obturated using MSUA showed the lowest percentage volume of voids compared to the other groups (*P* < 0.05). The mean volume percentage of voids in the MS group was statistically lower than that in the LC4 group (*P* < 0.003), but there was no significant difference between the LC2 group with LC4 and MS groups (*P* > 0.05).

In the apical third, the canals obturated using MSUA showed the lowest percentage volume of voids compared to the other groups (*P* < 0.05). The mean volume percentage of voids in the MS group was statistically lower than that in the LC4 group (*P* < 0.01), but there was no significant difference between the LC2 group with LC4 and MS groups (*P* > 0.05).

**Figure 2:**
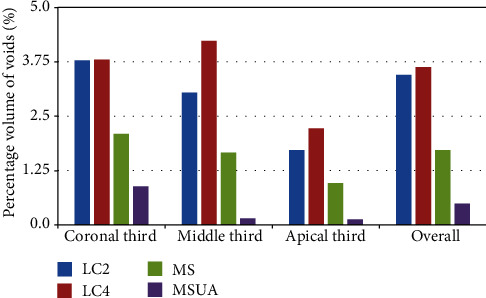
Percentage volume of voids in the different thirds and in the whole canal (overall) using lateral condensation using 0.02 tapered master cone (LC2), lateral condensation using 0.04 tapered master cone (LC4), matched single-cone technique (MS), and matched single cone-mediated ultrasonic activation (MSUA).

The summary of the mean percentage volume of voids associated with each obturation technique is depicted in Figure 2.

**Figure 3:**
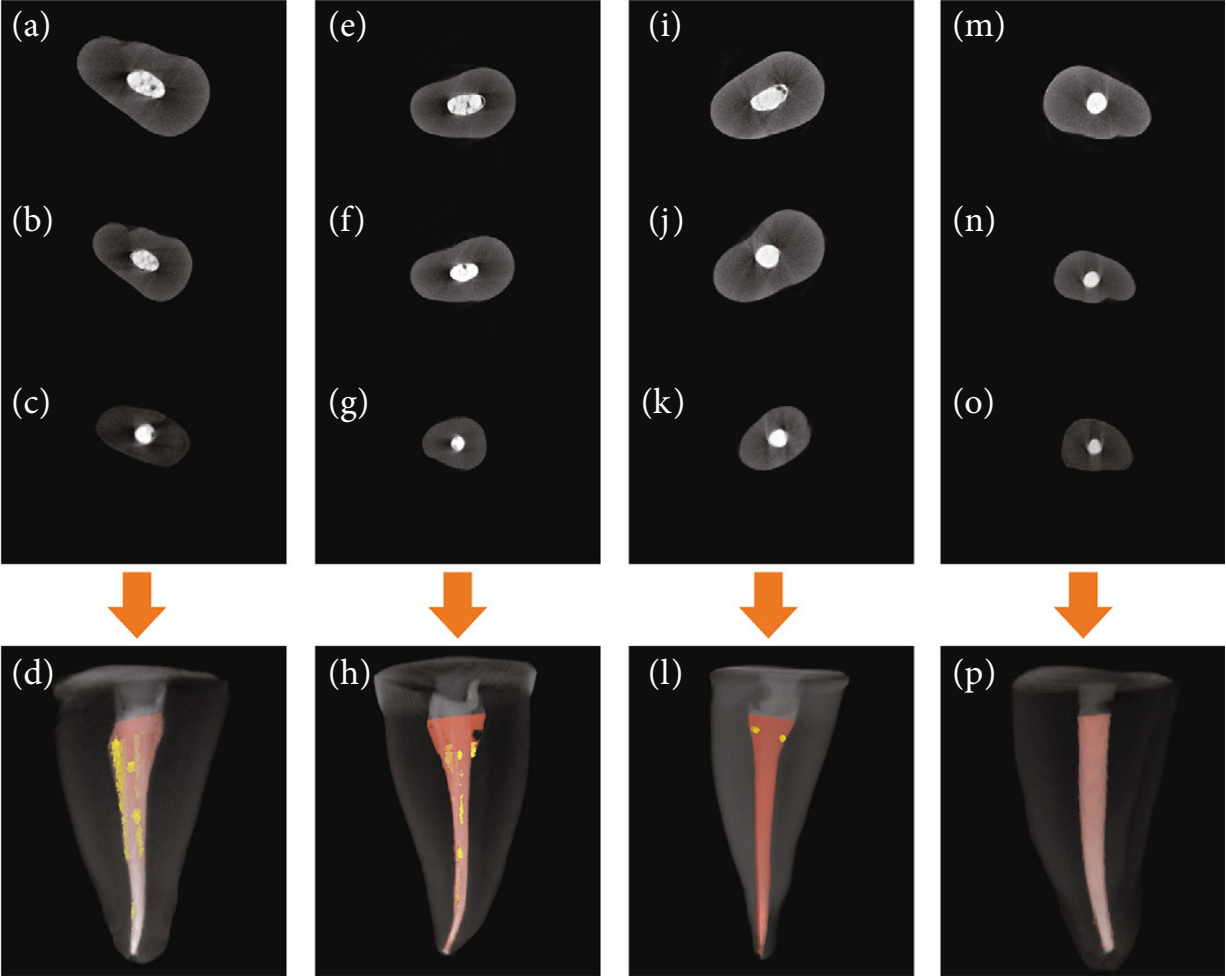
2D axial cross-sections and 3D reconstruction in the root canal filled teeth using different obturation techniques ((a–d) lateral condensation using 0.02 master cone; (e–h) lateral condensation using 0.04 master cone; (i–l) matched single-cone technique; (m–p) matched single cone-mediated ultrasonic activation). In the 3D figures, filling materials (gutta-percha/sealer) are in the orange color, and gaps/voids in the filling materials are in the yellow color.

2D slices of axial cross-sections of obturated root canals and 3D reconstructions from specimens are shown in Figure 3.

## 4. Discussion

Void-free filling of root canals is known to be associated with the higher success rate of primary root canal treatment [19]. In the present study, four different obturation techniques were evaluated using micro-CT in the mostly round root canals. Oval root canals are not generally suggested to be filled using the MS technique. It is due to the higher volume of sealer thickness in oval canals which can result in the dimensional shrinkage of the sealer during setting [4]. Historically, in endodontics, the thinner layer of the root canal sealer is preferred to decrease the potential of leakage due to sealer shrinkage during setting and dissolution over time [2].

Lateral condensation technique is known to be the most taught and utilized method of obturation worldwide [4]. Regarding this technique, a few studies have evaluated the influence of the taper of master cone on the quality of root canal filling. Bidar et al. utilized a fluid filtration method and did not find any significant difference when using 0.02 or 0.04 tapered GP as the master cone [20]. Similar results were seen in the study of Nagas et al. with the use of dye penetration method [21]. However, based on the results of our study, lateral condensation technique using 0.04 tapered master cone showed the highest (but not significant) mean percentage volume of voids, especially in the medial and apical third. It could possibly be attributed to the more shallow penetration of the spreader apically as the taper of the master cone increases. Allison et al. found that apical leakage is considerably lower when the spreader tip could be inserted within 1 mm of the working length compared to when the distance between the spreader tip and working length was greater [22]. It should be noted that in our study, when 0.04 master cone was used, the spreader could not penetrate more than 2-3 mm from the working length. On the other hand, when 0.02 master cone was used, the spreader could penetrate about 1 mm from the working length.

In general, lateral condensation technique either with 0.02 or 0.04 tapered master cone had significantly higher percentage volume of void compared to MS and MSUA. It can be attributed to the homogenous core and tightly fit F3 master GP point to the mostly round root canal walls used in this study. Logically, it may seem that the varying taper design of the F3 file and its intended preparation of the canal with a noncontinuous taper might affect the quality of obturation in the lateral condensation technique in the present study. However, the amount of spreader penetration is the key factor that affects the quality of obturation in this technique [22]. F3 file has a larger degree of taper (9%) over the first 3 mm from the D0 tip and a medium taper (5%) at the coronal end. Given that conventional coronal shaping instruments typically enlarge the coronal portion of the canals to coronal taper of 11% (e.g., when using S1), the design of the F3 file inherently does not play a role in diminishing the quality of the obturation. Therefore, the final diameter of the shaped root canal is sufficient for a deep and easy penetration of a spreader with 2% taper. In a study by Schäfer et al., it was also addressed that the type of instrument used for canal preparation has no effect on the quality of lateral condensation obturation [23].

When using the MS technique, it is suggested to take advantage of sealers with high flowability and adaptation to the irregularities of the root canal system [9]. Although bioceramic sealers are considered to be suitable for this technique, several studies have evaluated the use of epoxy resin-based sealers as well. Some authors have attempted to improve the distribution ability of resin-based sealers in the root canal [24, 25]. In these studies, the direct application of the ultrasound power was utilized. Guimarães et al. carried out this procedure by connecting a NiTi spreader to an ultrasonic device to distribute the sealer in the root canal [25]. This study evaluated 4 different epoxy resin-based sealers including AdSeal, AH Plus, Acroseal, and Sealer 26. Although this method had positive outcomes, it may carry the risk of instrument separation and also the unintentional potential for dentin removal from the root wall. In our study, indirect use of the ultrasound energy (only for 3 s) was utilized; thus, there would be no risk for iatrogenic errors. Moreover, the indirect distribution of the sealer via ultrasonic may lead to less overextrusion of the sealer from the apical foramen compared to the direct application of ultrasonic energy.

In the present study, AdSeal, an epoxy resin-based sealer, was used. There are reports in the literature about radiopacity values and physical properties of AdSeal, validating it as a relevant root canal sealing agent [26, 27]. In a study by Marciano et al., no statistical differences were found for adaptation, percentage of voids, solubility, flow, and film thickness when using AdSeal, AH Plus, or Acroseal [27]. From these studies it can be inferred that other epoxy resin-based sealers with similar physical characteristics can be used with the MSUA technique. However, further studies are needed to provide definitive evidence for this.

Orhan et al. evaluated the threshold values for detecting root canal filling voids in micro-CT and nano-CT images [28]. Except for the voxel size of 16.73 *μ*m, the values of the different nano-CT voxel sizes did not significantly differ from those of the micro-CT (5.2, 8.1, and 11.2 *μ*m). The authors concluded that a voxel size of 11.2 *μ*m can be considered a reliable cutoff value for the assessment of root canal filling voids in micro-CT imaging. It should be noted that in this study, we used a micro-CT scanner with a resolution of 19 *μ*m. Therefore, it seems valid to assume that there were some minute voids undetected by micro-CT in our study.

Using MS, GP cone relies on the original shape of the canal and the ability to create a tapered and circular preparation [7]. Therefore, the MS technique might be indicated and suitable for round canals [7, 15, 29]. When using such a technique, the practitioner can improve the even distribution of sealer by applying an indirect ultrasonic energy (i.e., MSUA).

This was an ex vivo study using micro-CT analysis to evaluate the volume of voids and gaps in the root canal fillings. It is not still clear if such an evaluation can be directly related to microbial leakage. Moreover, long-term stability and dissolution of the sealers should be taken into account for the application of this technique for clinical uses. Further clinical studies are necessary to confirm the clinical efficacy of the application of MS and MSUA techniques using epoxy-resin sealers. It should be taken into account that based on the higher volume of the sealer used in the single-cone obturation method, indeed, it is a sealer-based technique. Therefore, the long-term volume stability of the sealer is an important factor to consider and this technique should be used with caution until further studies can evaluate and provide definitive evidence regarding the sealer properties after obturation is complete.

## 5. Conclusions

MSUA, significantly, showed the least root canal filling voids amongst all the obturation techniques studied. MSUA can be considered an effective method for the filling of the round root canals. Lateral condensation using 0.04 tapered master cone showed the highest percentage volume of voids in the apical third of the canal. In general, lateral condensation using either 0.02 or 0.04 tapered master cones had significantly the highest volume percentage of voids amongst the experimental groups.

## Figures and Tables

**Table 1:** Means and standard deviations of percentage volume of voids (%) in the root canal filled teeth using different obturation techniques.

	Coronal third	Middle third	Apical third	Overall
LC2	3.78 ± 0.58^a^	3.04 ± 0.81^ab^	1.72 ± 0.65^ab^	3.45 ± 0.39^a^
LC4	3.8 ± 0.44^a^	4.23 ± 0.93^a^	2.22 ± 0.48^a^	3.63 ± 0.41^a^
MS	2.09 ± 0.68^b^	1.66 ± 1.2^b^	0.96 ± 0.57^b^	1.72 ± 0.59^b^
MSUA	0.81 ± 0.27^c^	0.15 ± 0.07^c^	0.13 ± 0.08^c^	0.49 ± 0.16^c^

^a,b,c^Different letters in each column indicate significant difference (*P* < 0.05). LC2: lateral condensation using 0.02 tapered master cone; LC4: lateral condensation using 0.04 tapered master cone; MS: matched single-cone technique; MSUA: matched single cone by ultrasonic activation.

## Data Availability

The data used to support the findings of this study are included within the article.

## Additional Points

(1) Matched single-cone technique with or without ultrasonic activation can efficiently fill the round root canals. However, using ultrasonic activation as a conjunct can prominently increase the quality of the filling. (2) Matched single cone-mediated ultrasonic activation can facilitate the obturation procedure in hard-to-reach teeth with round canals. (3) Epoxy-resin sealers can be proper when using matched single cone-mediated ultrasonic activation.
